# NBS superfood: a promising adjunctive therapy in critically ill ICU patients with omicron variant of COVID-19

**DOI:** 10.1186/s13568-024-01690-8

**Published:** 2024-03-24

**Authors:** Mehrdad Mosadegh, Aref Khalkhali, Yousef Erfani, Manije Nezamdoost, Seyyed Hamid Hashemi, Farid Azizi Jalilian, Nastaran Ansari, Shahab Mahmoudvand, Mojgan Mamani, Elham Abdoli, Razieh Amini, Gholamreza Kalvandi

**Affiliations:** 1 Department of Pathobiology, School of Public Health, Tehran University of Medical Sciences, Tehran, Iran; 2 Department of Science, Faculty of Biology, Islamic Azad University, Mashhad, Iran; 3 Department of Medical Laboratory Sciences, School of Allied Medical Sciences, Tehran University of Medical Sciences, Tehran, Iran; 4 Department of Internal Medicine, Division of Infectious Disease, Farabi Hospital, Social Security Organization, Mashhad, Iran; 5 Department of Infectious Diseases, Faculty of Medicine, Hamadan University of Medical Sciences, Hamadan, Iran; 6 Department of Virology, Faculty of Medicine, Hamadan University of Medical Sciences, Hamadan, Iran; 7 Research Center for Molecular Medicine, Hamadan University of Medical Sciences, Hamadan, Iran; 8 Department of Pediatrics, Faculty of Medicine, Hamadan University of Medical Sciences, Hamadan, Iran

**Keywords:** Nutrition bio-shield, SARS-CoV-2 Omicron variant, COVID-19, Complementary therapies, Respiratory distress syndrome

## Abstract

This clinical trial aimed to assess the impact of Nutrition Bio-shield superfood (NBS) on clinical status among critically ill ICU patients suffering from acute respiratory distress syndrome (ARDS) due to the Omicron variant of COVID-19. A total of 400 patients with confirmed Omicron-related ARDS were randomly assigned to either the intervention group (n = 200) or the control group (n = 200). Patients in the intervention group received 1.5 g of NBS powder daily for 2 weeks in addition to standard antiviral treatment, while the control group received a placebo alongside standard antiviral therapy. Serum samples were collected from all patients in both groups, and various clinical and laboratory parameters, including ESR, CRP, D-Dimer, CPK, WBC count, lymphocyte count, and lymphocyte percentage, were measured using established methodologies. Following a 14-day intervention period, the intervention group exhibited a significant reduction in mean serum levels of CRP (15.39 vs. 48.49; P < 0.001), ESR (14.28 vs. 34.03; P < 0.001), D-Dimer (485.18 vs. 1009.13; P = 0.001), and CPK (68.93 vs. 131.48; P < 0.001) compared to the control group. Conversely, a significant increase was observed in the mean serum levels of lymphocytes (1537.06 vs. 1152.60; P < 0.001) in the intervention group after 14 days of treatment compared to the control group. The remarkable reduction in inflammatory markers and mortality rates observed with NBS supplementation alongside standard antiviral treatment underscores its crucial role in mitigating inflammation and achieving an important milestone in the fight against COVID-19.

## Introduction

Severe acute respiratory syndrome coronavirus 2 (SARS-CoV-2), initially documented on December 31, 2019, has been identified as the etiological agent responsible for the onset of the infectious ailment recognized as coronavirus disease 2019 (COVID-19) (Azizi Jalilian et al. 2022). COVID-19, having disseminated globally, attained the status of a global pandemic as officially declared by the World Health Organization (WHO) on March 11, 2020 (Weidmann et al. 2021; Oristrell et al. 2022). According to the data released by the WHO, as of February 4, 2024, the cumulative count of confirmed COVID-19 cases stands at 774,593,066, accompanied by a total of 7,028,881 recorded fatalities. Furthermore, as of August 5, 2023, a cumulative total of 13,492,225,267 vaccine doses has been globally administered (https://covid19.who.int/). In Iran, as of August 5, 2023, a cumulative total of 155,441,243 vaccine doses has been administered. Nonetheless, despite widespread vaccination efforts, from January 3, 2020, to February 18, 2024, the COVID-19 pandemic has persisted with an unsettling toll, registering over 7,626,230 confirmed cases and surpassing 146,785 recorded fatalities (https://covid19.who.int/region/emro/country/ir).

Research findings have elucidated that individuals aged over 65 years, those afflicted by chronic obstructive pulmonary disease, diabetes mellitus, chronic renal disease, malignant tumors, hyperlipidemia, hypertension, and post-transplantation immunodeficiency, constitute a cohort vulnerable to the severe manifestation of the disease (Gadotti et al. 2020; Azimi et al. 2021).

Patients afflicted by SARS-CoV-2 typically present with dual clinical profiles. In the majority, constituting 75 to 80% of cases, the disease manifests as a spectrum of symptoms spanning mild to moderate, encompassing manifestations such as fever, chills, fatigue, cough, sore throat, and alterations in taste and/or olfaction. Conversely, a subset comprising 10 to 15% of patients manifests a severe form of the ailment (Leulseged et al. 2021; Legacy et al. 2022). In the severe form of the disease, patients commonly exhibit notable abnormalities, including pulmonary infiltrates, multi-organ dysfunction, and declining oxygen saturation levels. Typically, individuals with these manifestations necessitate hospitalization in tertiary care facilities (Azimi et al. 2021). Furthermore, it has been observed that in the context of severe COVID-19 pathology, SARS-CoV-2 can instigate a cascade of events, characterized by hyperactivation of the immune system, disproportionate release of proinflammatory cytokines, heightened oxidative stress, and the activation of pro-coagulatory factors (Mortaz et al. 2021).

Laboratory findings have demonstrated that patients experiencing the severe form of COVID-19 infection exhibit elevated neutrophil counts and increased levels of various factors, including serum C-reactive protein (CRP), interleukin-1 beta (IL-1β), IL-2, IL-6, IL-7, IL-8, IL-10, and IFN gamma associated with TH1 response. Conversely, their blood samples indicate a significant reduction in platelet and lymphocyte counts. It is widely acknowledged that the immune response to SARS-CoV-2, along with the secretion of inflammatory cytokines, plays a pivotal role in the progression of the infectious disease (Speakman et al. 2021; Mosadegh et al. 2022a, b).

Dietary supplements play a valuable role in modulating and supporting the functioning of the immune system. Extensive research has shown that vitamins, minerals, and herbs have the potential to mitigate the duration and severity of viral infections, including influenza (Beigmohammadi et al. 2021; Gönen et al. 2021). The Nutrition Bio-Shield (NBS Organik®, İstanbul, Turkey) powder is designated with an approved identification number (006633-14.11.2019) and is categorized as a natural herbal supplement known for its immune system-enhancing properties (Bayat et al. 2014). NBS primarily derives from wheat germ and encompasses a diverse array of essential vitamins, including A, B1–B3, B5, B6, B9, C, D, K, and a complement of vital minerals such as potassium, manganese, magnesium, phosphorus, sulfur, boron, calcium, iron, zinc, copper, as well as essential fatty acids like omega-6 and omega-9, in addition to other macro and micro molecules (Azizi Jalilian et al. 2022; Mosadegh et al. 2022a, b).

Currently, a variety of vaccines are available for the prevention and treatment of COVID-19 infection (Le et al. 2020, Tzenios et al. 2023). However, in response to the emergence of novel variants of the SARS-CoV-2 virus, the imperative task of designing and conducting clinical trials to identify effective and innovative therapeutic interventions becomes increasingly vital. The aim of this clinical trial study is to assess the impact of NBS powder on the clinical condition and inflammatory markers in critically ill intensive care unit (ICU) patients afflicted with acute respiratory distress syndrome (ARDS) attributed to the Omicron variant of COVID-19.

## Materials and methods

### Ethics approval and trial registration

The study protocols adhered to the principles outlined in the Helsinki Declaration, with all procedures receiving official validation from the Ethics Committee of Hamadan University of Medical Sciences (Protocol Number: IR.UMSHA.REC.1401.1043; Approval Date: 2023-03-04). Additionally, the study’s protocol has been registered in the Iranian Registry of Clinical Trials (IRCT), accessible at www.irct.ir (IRCT Registration Number: IRCT20230116057135N2; Registration Date: 2023-04-05).

### Study design and participants

**Fig. 1:**
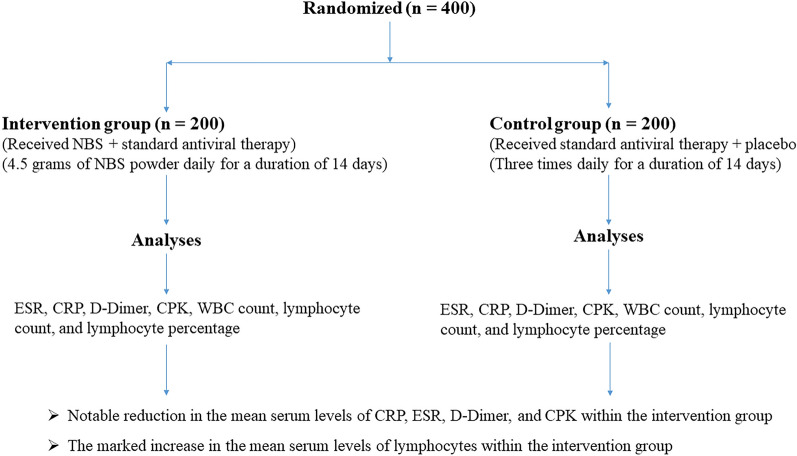
Consort flow chart

The current investigation employed a rigorous double-blind, randomized clinical trial design conducted at Farabi Hospital in Mashhad, Iran. The objective of this study was to assess the impact of NBS on the clinical condition and mortality rate among critically ill ICU patients suffering from ARDS attributed to the Omicron variant of COVID-19. The study's participants were exclusively drawn from the pool of individuals admitted to the ICU at Farabi Hospital, each of whom had received a definitive diagnosis of the Omicron variant of COVID-19. A total of 400 patients were enrolled in the study, with 200 patients assigned to the intervention group and another 200 to the control group (Fig. 1). The informed written consent was meticulously obtained from all enrolled patients, ensuring their voluntary participation in the study.

### Inclusion criteria

Inclusion criteria for the current study encompassed patients who fulfilled the following conditions: (1) willingness to participate, as demonstrated by the signing of written informed consent, (2) a confirmed diagnosis of the Omicron variant of COVID-19, accompanied by ARDS, (3) admission to the ICU setting, (4) absence of underlying comorbidities, and (5) inclusion of both male and female patients. The definitive confirmation of the Omicron variant of COVID-19 was established through a two-tiered diagnostic process involving Reverse Transcriptase-Polymerase Chain Reaction (RT-PCR) assay, which included the retrieval of RT-PCR Ct values, and subsequent genome sequencing analysis.

### Exclusion criteria

Patients were excluded from participation in the present study based on the following criteria: (1) prior involvement in other clinical trial studies, (2) non-consent and absence of signed informed consent, (3) testing negative for the Omicron variant of COVID-19, (4) manifestation of ARDS attributed to alternative viral infections, such as human immunodeficiency virus, Hepatitis C, Hepatitis B, or other common respiratory viruses, (5) presence of underlying medical conditions or history of solid organ or hematological transplantation, (6) pregnancy, and (7) consumption of supplementary substances within the three-month period preceding the commencement of the study.

### Interventions and randomization

The study enrolled a total of 400 patients who presented with ARDS and received a confirmed diagnosis of the Omicron variant of COVID-19. Employing a randomized allocation method, all patients were subsequently divided into two distinct groups: (1) the intervention group (n = 200) and (2) the control group (n = 200).

In the intervention group, patients received a specific treatment regimen comprising the NBS superfood in addition to standard antiviral therapy. This entailed the administration of 4.5 g of NBS powder daily, distributed across three equal doses: 1.5 g in the morning (6 a.m. to 12 p.m.), 1.5 g in the afternoon (12 p.m. to 6 p.m.), and 1.5 g in the evening (6 p.m. to 12 a.m.), administered over a duration of 2 weeks. Patients allocated to the control group received standard antiviral therapy alongside a placebo. The placebo was administered three times daily for a duration of 14 days. This regimen was devoid of any active ingredients, serving as an inert comparator. The standard antiviral treatment protocol encompassed the administration of Kaletra (Lopinavir + Ritonavir) (AbbVie Inc., North Chicago, Illinois, U.S.A) in combination with Hydroxychloroquine (Oxiklorin, Elyson Pharmaceutical Co. Ltd., Seoul, Korea). It is pertinent to note that the ICU specialists were informed of the intervention allocation for both study groups, while patients enrolled in both the intervention and control groups as well as nurses remained blinded to their respective treatment assignments.

### Clinical and laboratory biomarkers measurements

Blood samples, totaling 10 ml (comprising 5 ml at the outset of the study and an additional 5 ml following the intervention), were meticulously collected from all patients enrolled in both the intervention and control groups. These samples were collected to facilitate the assessment of clinical and laboratory parameters. All blood samples were acquired utilizing standard coagulation tubes and subsequently subjected to centrifugation at 10,000 revolutions per minute (rpm) for a duration of 10 min to facilitate serum isolation. Subsequently, the quantification of clinical and laboratory parameters, including Erythrocyte Sedimentation Rate (ESR), serum CRP, D-Dimer (a fibrin degradation product), Creatine Phosphokinase (CPK), White Blood Cell (WBC) count, lymphocyte count, and lymphocyte percentage, was executed through the application of established kits and methodologies. These assessments were conducted both at baseline (prior to intervention initiation) and on the 14th day following the intervention.

### Statistical analysis

All clinical attributes and laboratory findings were meticulously incorporated into SPSS version 23.0 (SPSS Inc., Chicago, IL, USA). Comprehensive data analysis was conducted employing a battery of statistical tests, including independent samples t-test, paired t-test, and the chi-square test, as appropriate for the specific data characteristics and objectives of the study.

## Results

### Comparison of clinical characteristics and laboratory results in both control and intervention groups

In the intervention group, the mean age of all patients was 51.33 ± 14.84 years, with an age range spanning from 22 to 93 years. Conversely, in the control group, the median age was 51.74 ± 13.01 years, ranging from 26 to 83 years.

Table 1 provides a comprehensive summary of the clinical characteristics and laboratory results of all patients in both the intervention and control groups at both the baseline and the 14-day post-intervention time points. Significant statistical differences were observed in the levels of CRP (P < 0.001), ESR (P < 0.001), D-Dimer (P < 0.001), and CPK (P < 0.001) in the control group 14 days following the administration of placebo alongside standard antiviral treatment. The findings indicated an increase in CRP, ESR, D-Dimer, and CPK levels at the 14-day post-standard antiviral treatment interval.

**Table 1:** Summary of the clinical characteristics and laboratory results of all patients in both the intervention and control groups at both the baseline and the 14-day post-intervention

Variables (unit)	Control group (baseline) N = 200			Control group (after intervention) N = 200			P-value
	Mean	Std. deviation	Std. error mean	Mean	Std. deviation	Std. error mean	
CRP (mg/L)	37.6350	24.17548	1.70946	48.4850	32.60113	2.30525	0.000^**a**^
ESR (mm/h)	28.0000	16.52408	1.16843	34.0350	16.07938	1.13698	0.000^**a**^
D-Dimer (ng/ml)	759.2600	503.91503	35.63217	1009.1300	699.12253	49.43543	0.000^**a**^
CPK (U/L)	97.4550	81.17719	5.74009	131.4800	102.95103	7.27974	0.000^**a**^
Lymphocyte count	1206.3800	374.83656	26.50495	1152.5950	551.33028	38.98494	0.035^**a**^

Variables (unit)	Intervention group (baseline) N = 200			Intervention group (after intervention) N = 200			P-value
	Mean	Std. deviation	Std. error mean	Mean	Std. deviation	Std. error mean	
CRP (mg/L)	40.7950	27.96575	1.97748	15.3850	11.54745	0.81653	0.000^**a**^
ESR (mm/h)	26.4550	15.29732	1.08168	14.2750	8.09674	0.57253	0.000^**a**^
D-Dimer (ng/ml)	807.7950	438.69631	31.02051	485.1750	279.05622	19.73225	0.000^**a**^
CPK (U/L)	100.1150	84.44416	5.97110	68.9300	57.35804	4.05583	0.000^**a**^
Lymphocyte count	1135.5350	362.60799	25.64026	1537.0600	469.04103	33.16621	0.000^**a**^

C-Reactive Protein (CRP), Erythrocyte Sedimentation Rate (ESR), D-Dimer (a fibrin degradation product), *Creatine Phosphokinase* (CPK), N: number of samples

^a^Paired t-test

In contrast, our analyses unveiled a substantial decrease in the mean levels of CRP (P < 0.001), ESR (P < 0.001), D-Dimer (P < 0.001), and CPK (P < 0.001) within the intervention group, observed 14 days subsequent to the administration of NBS alongside standard antiviral treatment. Furthermore, a noteworthy increase in the mean lymphocyte count (P < 0.001) was documented 14 days post-consumption of NBS superfood.

### Comparison of variables in both control and intervention groups at the baseline and after a 14-days interval

Table 2 provides a concise summary and comparative analysis of the clinical attributes and laboratory results for all patients enrolled in both the intervention and control groups, both at the study’s outset and 14 days following the intervention. The comparative analysis of the investigated variables between the control and intervention groups demonstrated a notable reduction in the mean serum levels of CRP (15.39 versus 48.49; P < 0.001), ESR (14.28 versus 34.03; P < 0.001), D-Dimer (485.18 versus 1009.13; P = 0.001), and CPK (68.93 versus 131.48; P < 0.001) at the 14-day post-intervention juncture within the intervention group, in stark contrast to the control group. Conversely, a marked increase was observed in the mean serum levels of lymphocytes (1537.06 versus 1152.60; P < 0.001) 14 days following the intervention within the intervention group, compared to the control group.

**Table 2:** Comparison of the variables in both control and intervention groups at both the baseline and the 14-day post-intervention

Variables	Groups	N	Mean	Std. deviation	Std. error mean	P-value
Group statistics						
CRP (baseline)	Intervention	200	40.80	27.97	1.98	0.227^a^
	Control	200	37.64	24.18	1.71	
CRP (after intervention)	Intervention	200	15.39	11.55	0.82	0.000^a^
	Control	200	48.49	32.60	2.31	
ESR (baseline)	Intervention	200	26.46	15.30	1.08	0.332^a^
	Control	200	28.00	16.52	1.17	
ESR (after intervention)	Intervention	200	14.28	8.10	0.57	0.000^a^
	Control	200	34.03	16.08	1.14	
D-Dimer (baseline)	Intervention	200	807.80	438.70	31.02	0.305^a^
	Control	200	759.26	503.92	35.63	
D-Dimer (after intervention)	Intervention	200	485.18	279.06	19.73	0.000^a^
	Control	200	1009.13	699.12	49.44	
CPK. (baseline)	Intervention	200	100.12	84.44	5.97	0.748^a^
	Control	200	97.46	81.18	5.74	
CPK (after intervention)	Intervention	200	68.93	57.36	4.06	0.000^a^
	Control	200	131.48	102.95	7.28	
Lymphocyte (baseline)	Intervention	200	1135.54	362.61	25.64	0.055^a^
	Control	200	1206.38	374.84	26.50	
Lymphocyte (after intervention)	Intervention	200	1537.06	469.04	33.17	0.000^a^
	Control	200	1152.60	551.33	38.98	

C-Reactive Protein (CRP), Erythrocyte Sedimentation Rate (ESR), D-Dimer (a fibrin degradation product), *Creatine Phosphokinase* (CPK), N: number of samples

^a^Independent samples t-test

We did not find significant differences in the mean of serum levels of CRP (P = 0.227), ESR (P = 0.332), D-Dimer (P = 0.305), and CPK (P = 0.748) at baseline between intervention and control groups.

### The effects of NBS consumption on surveyed variables

To evaluate the association between the percentage of patients transitioning from abnormal to normal values for various parameters within both the intervention and control groups, we employed the Chi-square (χ^2^) test. Our analysis yielded statistically significant differences in the proportions of patients achieving this transition between the two cohorts. For CRP, it is noteworthy that 27.5% of patients in the intervention group and a mere 17.5% in the control group exhibited a transition from abnormal to normal values, with a remarkably low P-value of 0.000. In the case of ESR, a substantial disparity is evident, as 43% of patients in the intervention group and only 5% in the control group experienced a transition from abnormal to normal values (P = 0.000). With regard to D-Dimer, 42.5% of patients in the intervention group and a mere 8.3% in the control group successfully achieved a transition from abnormal to normal values, signifying a statistically significant difference with a P-value of 0.000. Lastly, for CPK, 18% of patients in the intervention group and merely 10.5% in the control group demonstrated a transition from abnormal to normal values, with a significant P-value of 0.000.

### The mortality rate in both the control and intervention groups

Overall, the study revealed mortality rates of 31% (n = 62/200) and 8.5% (n = 17/200) within the control and intervention groups, respectively, with a statistically significant disparity (P = 0.001), underscoring the substantial impact of NBS consumption in reducing patient mortality, as delineated in Table 3. Furthermore, upon conducting a more detailed analysis within the intervention group, it was ascertained that 10.3% (n = 9/87) and 7.1% (n = 8/113) of deceased patients were male and female, respectively. Importantly, this analysis did not reveal any statistically significant differences (P = 0.412) between the genders in terms of mortality outcomes. Conversely, within the control group, it was observed that 25.9% (n = 21/81) and 34.55% (n = 41/119) of deceased patients were female and male, respectively, with no statistically significant distinction noted (P = 0.201). Cumulatively, the results indicated that among all patients in both the control and intervention groups, 38.7% (n = 67/173) of deceased patients were aged 50 years or older, and this difference was statistically significant (P = 0.001).

**Table 3:** The mortality rate in control and intervention groups

	Expire		Total	P-value
	Yes	No		
Groups				
Control				0.000^a^
Count	62	138	200	
%	31.0%	69.0%	100.0%	
Intervention				
Count	17	183	200	
%	8.5%	91.5%	100.0%	
Total				
Count	79	321	400	
%	19.8%	80.3%	100.0%	
Gender (total)				
Female				0.418^a^
Count	30	138	168	
%	17.9%	82.1%	100%	
Male				
Count	49	183	232	
%	21.1%	78.9%	100%	
Total				
Count	79	321	400	
%	19.8%	80.2%	100%	
Gender (control group)				
Female				0.201^a^
Count	21	60	81	
%	25.9%	74.1%	100%	
Male				
Count	41	78	119	
%	34.55	65.5%	100%	
Total				
Count	62	138	200	
%	31%	69%	100%	
Gender (intervention group)				
Female				0.412^a^
Count	9	78	87	
%	10.3%	89.7%	100%	
Male				
Count	8	105	113	
%	7.1%	92.9%	100%	
Total				
Count	17	183	200	
%	8.5%	91.5%	100%	
Age groups				
< 50 years old				0.000^a^
Count	12	215	227	
%	5.3	94.7	100	
≥ 50 years old				
Count	67	106	173	
%	38.7	61.3	100	
Total				
Count	79	321	400	
%	19.8	80.3	100	

^a^Chi-square test

## Discussion

The challenges of treating COVID-19 in the face of different variants involve considerations of reduced vaccine effectiveness, increased transmissibility, potential resistance to treatments, and the need for ongoing surveillance and adaptation of countermeasures to combat the evolving virus(Planas et al. 2021; Tegally et al. 2021; Wang et al. 2021). These challenges highlight the importance of a multidisciplinary approach involving research, public health measures, and healthcare interventions to manage the pandemic effectively. The primary focus of this study, as well as many recent research endeavors, is to investigate the efficacy of suggested primary and adjunctive therapies as alternatives to target-specific treatments, given the reasons outlined above.

Five distinct variants (Alpha, Beta, Gamma, Delta, and Omicron) of SARS-CoV-2 have been recognized as significant global public health concerns (Meo et al. 2021). The most recent variant, Omicron, was initially identified on November 24, 2021, in South Africa and Botswana, and was subsequently designated as a novel variant by the WHO (Fan et al. 2022). The elevated number of mutations present in the Omicron variant of SARS-CoV-2 has the potential to impact the efficacy of existing vaccines and treatment protocols (Kandeel et al. 2022). As highlighted earlier, the Omicron variant poses a potential challenge to global endeavors aimed at controlling SARS-CoV-2 infections. Consequently, there is a compelling need for the meticulous design and execution of comprehensive clinical trial studies to identify effective and innovative therapeutic agents.

The objective of the present study was to assess the efficacy of NBS powder, a natural supplement, on the clinical condition and inflammatory markers of critically ill ICU patients suffering from ARDS attributed to the Omicron variant of COVID-19. Prior to the study, the definitive diagnosis of the Omicron variant of COVID-19 had been established through molecular-based testing and sequencing assays.

The comparative analysis within the control group exhibited a notable and statistically significant augmentation in the mean serum levels of CRP, ESR, D-Dimer, and CPK at the 14-day post-intervention, compared to the baseline. In the presence of inflammation, hepatic synthesis of CRP occurs, leading to its release into the bloodstream. Additionally, the ESR serves as an indicator of systemic inflammation within the circulatory system. The manifestation of pain, swelling, and erythema in affected tissues can be attributed to the production of CRP and elevation in ESR levels, commonly observed in the context of chronic diseases or autoimmune disorders (Sproston and Ashworth 2018). Inflammation can be triggered by a spectrum of conditions, encompassing bacterial or viral infections, arthritic disorders, vasculitis, and inflammatory bowel disease (Ahmed 2011). Elevated results in both the CRP and ESR tests are indicative of systemic inflammation within the body (Sproston and Ashworth 2018). Furthermore, D-Dimer has emerged as a noteworthy prognostic indicator for assessing the severity and mortality risk associated with COVID-19. Thromboembolic complications have been associated with elevated D-dimer levels, a phenomenon attributed not only to such complications but also to acute lung injury in the context of COVID-19 infection (Yao et al. 2020). CPK, an enzyme, naturally occurs in the body and is predominantly localized in the heart, brain, and skeletal muscle tissues. Elevated blood levels of CPK are indicative of injury or stress affecting the heart, brain, or muscle tissues (Kuwahara et al. 2010). Alterations in lymphocyte levels, characterized by increases and decreases, are clinically referred to as lymphocytosis and lymphocytopenia, respectively (Shanafelt et al. 2009). The findings of this study unveiled a notable decrease in mean serum lymphocyte levels at the 14-day post-standard antiviral treatment assessment within the control group, in comparison to the baseline measurements. Lymphocytes represent a pivotal component of the body’s immune system, tasked with combating foreign bacteria and viruses. Deviations in lymphocyte levels within the bloodstream, whether elevated or reduced, may bear relevance to underlying inflammatory processes (Jafarzadeh et al. 2021).

Conversely, our analyses unveiled a substantial decrease in the mean levels of CRP, ESR, D-Dimer, and CPK within the intervention group at the 14-day interval subsequent to NBS powder administration alongside standard antiviral treatment. A comparative assessment of the surveyed variables between the control and intervention groups showcased a statistically significant reduction in mean serum levels of CRP, ESR, D-Dimer, and CPK, at the 14-day post-intervention period within the intervention group, in contrast to the control group. This study unequivocally underscores the potent impact of a 14-day regimen of NBS consumption on the meticulous modulation of pivotal inflammatory markers within the body. These results not only corroborate the robust efficacy of NBS but also elucidate its pivotal role in significantly attenuating the levels of CRP, ESR, CPK, and D-Dimer. This effect is instrumental in alleviating life-threatening conditions in patients, as evidenced by recent research findings (Mehta et al. 2020; Ruan et al. 2020; Zhou et al. 2020). Emerging evidence suggests that the mechanism of action underlying this profound effect lies in NBS's ability to mitigate the excessive release of proinflammatory cytokines, curtail oxidative stress, and counteract the activation of pro-coagulation factors, all of which contribute to the amelioration of inflammation and, consequently, improved disease severity and reduced mortality rates among COVID-19 patients. These compelling findings emphasize the potential of NBS as an invaluable adjunctive therapy in the battle against severe COVID-19.

The impact of NBS powder on disease severity and immune system function in COVID-19 patients has been investigated in several rigorous scientific studies. In a clinical trial conducted by Jalilian et al. in Iran, the impact of NBS on immune system function in COVID-19 patients was thoroughly investigated. Their study revealed that the consumption of NBS powder over a duration of 4 weeks yielded a significant influence on the levels of proinflammatory cytokines, particularly interleukin-6 (IL-6) and tumor necrosis factor α (TNF-α), in individuals afflicted with COVID-19 (Azizi Jalilian et al. 2022). Furthermore, Mosadegh et al. conducted a meticulously designed study investigating the impact of NBS superfood on a spectrum of laboratory biomarkers and disease severity in patients afflicted with COVID-19. The findings derived from their rigorously executed clinical trial unequivocally demonstrated a significant decrease in the mean serum levels of key parameters, including CRP, ESR, D-Dimer, lactate dehydrogenase (LDH), aspartate aminotransferase (SGOT), serum glutamic pyruvic aminotransferase (SGPT), alkaline phosphatase (ALP), WBC count, IL-6, and TNF-α, at the 14-day interval following intervention compared to baseline within the intervention group (Mosadegh et al. 2022a, b). Another investigation revealed that the NBS supplement, administered orally to rats with rheumatoid arthritis, significantly reduced serum levels of inflammatory markers such as ESR and CRP. Moreover, it effectively normalized rheumatoid factor (RF) levels, indicating its potential to alleviate RA symptoms. These findings corroborate the anti-inflammatory properties of NBS observed in clinical trials on COVID-19 patients, underlining its broader therapeutic potential across inflammatory conditions. Further research is warranted to solidify its clinical value, but these collective results emphasize the promise of NBS as an adjunctive therapy for inflammatory diseases (Mosadegh et al. 2022a, b). These results underscore the significant impact of NBS superfood in mitigating inflammation and ameliorating disease severity among COVID-19 patients, in alignment with the findings of the present scientific research. The evaluation of disease severity and inflammatory biomarkers among COVID-19 patients has been a subject of investigation by various researchers, who have explored the potential effects of herbal supplements and pharmacological interventions. In a study conducted by Emadi et al. in Iran, the oral administration of Imatinib in hospitalized adults with COVID-19 was meticulously examined to assess its efficacy and safety profile. Their research findings shed light on the notable impact of Imatinib on modulating inflammatory markers, such as IL-6, within the patient population afflicted with COVID-19 (Emadi et al. 2020). The impact of curcumin-piperine supplementation on inflammatory markers, oxidative stress, clinical parameters, and mortality rates among critically ill patients in the ICU afflicted with COVID-19 was rigorously investigated by Askari et al. in a study conducted in Iran. Their comprehensive findings illuminated the favorable effects of curcumin-piperine supplementation, manifesting in the normalization of key clinical indicators including fever, blood oxygen saturation, and respiratory rate. Additionally, their research indicated that the utilization of curcumin-piperine supplementation exerted significant influence on important clinical outcomes, encompassing the duration of ICU stay, mortality rates, as well as the levels of prominent biomarkers such as CRP, ESR, alanine aminotransferase (ALT), aspartate aminotransferase (AST), LDH, and creatinine (Askari et al. 2021). These findings contribute to the body of scientific knowledge about potential therapeutic interventions targeting inflammation and clinical outcomes in COVID-19 patients, aligning with the focus of the present study.

In a separate investigation conducted by Beigmohammadi et al. in Iran, the impact of vitamins A, B, C, D, and E on patient improvement and mortality rates within the context of COVID-19 was systematically evaluated. Their study illuminated the beneficial effects associated with vitamin consumption, particularly the modulation of key serum biomarkers including WBC count, CRP, ESR rate, IL-6, interferon-gamma (IFN-γ), and TNF-α (Beigmohammadi et al. 2020). These findings contribute valuable insights into the potential therapeutic role of supplementation in mitigating inflammatory responses and improving clinical outcomes among COVID-19 patients, aligning with the overarching objective of our investigation.

The findings of the present study have elucidated a notable disparity in mortality rates, whereby 31% of patients within the control group and 8.5% within the intervention group succumbed to the disease. This observation underscores the substantial impact of a 14-day regimen of NBS natural supplements in dramatically diminishing mortality rates among individuals afflicted with COVID-19. The mortality rate in the control group was substantially higher than in the intervention group, emphasizing the potential life-saving effects of NBS supplementation. While this study was not designed to explore the exact mechanisms underlying the reduction in mortality, it is plausible that the modulation of inflammation and immune response by NBS played a role in improving patient outcomes. One possible mechanism of NBS's action is its rich content of vitamins, minerals, and essential fatty acids. These nutrients are known to play roles in immune function and inflammation modulation. The diverse array of bioactive molecules in NBS may act synergistically to enhance the body’s defense mechanisms against the virus. Further research is needed to elucidate the specific mechanisms by which NBS exerts its effects.

The striking congruence observed in the outcomes of the present clinical trial and the antecedent investigations conducted by Jalilian et al. (Azizi Jalilian et al. 2022) and Mosadegh (Mosadegh et al. 2022a, b) attests to the robust and consistent efficacy of NBS superfood as an adjunctive therapeutic agent amidst the backdrop of distinct SARS-CoV-2 variants. It is of paramount significance to underscore the temporal context in which these studies transpired. While Jalilian’s inquiry centered on the alpha variant and Mosadegh’s exploration delved into the delta variant, the current trial unfolded amidst the tumultuous surge of the Omicron variant. The unwaveringly affirmative findings across this spectrum of viral strains lead us to a pivotal revelation: NBS’s mechanisms of action transcend the taxonomic boundaries of microorganisms, even in the face of the relentless mutational dynamics of emerging variants. This inherent adaptability delineates NBS as a unique and conceivably indispensable asset in the battle against infectious diseases, effectively countering the vexing challenges posed by the dynamic evolution of surface antigens in novel variants and the emergence of resistance phenomena. A foundational principle meriting recognition in the context of emerging viral variants resides in the potential attenuation of vaccine efficacy, frequently ascribed to the emergence of novel surface antigens and the subsequent evolution of antibody-resistant strains. These concerns have engendered a global reconsideration of therapeutic strategies, underscoring the urgency of innovative interventions. The extraordinary uniformity in the outcomes of the investigations by Jalilian, Mosadegh, and our present study underscores NBS as a beacon of optimism amidst these complex circumstances. It defies the conventional paradigms of pathogen-specific therapeutic modalities, presenting itself as a versatile and adaptable solution to the ceaselessly shifting landscape of viral variants. This profound attribute of NBS warrants further comprehensive examination and inquiry, as it may harbor the key to ameliorating the devastating consequences of emerging infectious diseases, fortifying our resilience in the face of the capricious forces of microbial evolution. As we confront the perpetual arms race between human scientific ingenuity and the adaptive abilities of viral entities, NBS emerges as a steadfast ally, offering a glimpse into the trajectory of pandemic preparedness and therapeutic innovation. The main limitation of the present study was budget limitation. The present study was a pilot study with a budget limitation. Therefore, we could not evaluate more biomarkers such as the levels of interleukins among included patients.

In conclusion, the present study has elucidated the capacity of NBS superfood supplement to effectively mitigate inflammatory markers in critically ill ICU patients afflicted by ARDS caused by the Omicron variant of COVID-19. These results underscore the potential utility of NBS superfood as an adjunct to standard antiviral treatments for inflammation reduction and the attenuation of mortality rates in COVID-19 patients.

## Data Availability

All data generated or analysed during this study are included in this published article.
